# Who should be considered for prostate artery embolization: revisiting the urologic guidelines based on results from over 300 patients with benign prostatic hyperplasia

**DOI:** 10.1186/s42155-026-00703-w

**Published:** 2026-10-23

**Authors:** Gabriel M. Knight, Ajay Varadhan, Elana Benishay, Delmarie M. Rivera-Rodríguez, Kimberly Jenkins, Elias Hohlastos, Riad Salem, Samdeep Mouli

**Affiliations:** Department of Radiology, Section of Vascular and Interventional Radiology, Northwestern University, Chicago, IL USA

**Keywords:** Prostate artery embolization, Benign prostatic hyperplasia, Guidelines, Urology

## Abstract

**Background:**

Prostate artery embolization (PAE) has emerged as a minimally invasive therapy which affords improvement in benign prostatic hyperplasia (BPH) symptoms and objective urinary parameters with a favorable adverse event (AE) profile. While PAE was recently included in the American Urological Association (AUA) Guidelines, guidance on its specific use-cases is limited. This study sought to answer remaining questions surrounding the safety and efficacy of PAE across different patient subpopulations where formal guidance for PAE is limited.

**Methods:**

Three hundred two patients with BPH underwent PAE. Key baseline characteristics were gathered. Patients were compared from baseline to follow-ups with regards to (1) objective parameters, including maximum urinary flow rate (Q_max_), post-void residual (PVR), and prostate specific antigen (PSA); (2) prostate volume; (3) BPH symptoms, including International Prostate Symptom Score (IPSS), IPSS Quality-of-Life (IPSS-QoL), and BPH Impact Index (BPH-II); (4) sexual symptoms, including the International Index of Erectile function (IIEF) and Male Sexual Health Questionnaire for Ejaculatory Function Short Form (MSHQ-EjD-SF); and (5) AEs. Additional sub-analyses were performed comparing the safety/efficacy of PAE across key sub-groups.

**Results:**

PAE afforded significant improvement (*p* < 0.05) in each subjective/objective BPH parameter and sexual function score at nearly all follow-up points. Specifically, IPSS improved by 12.3–13.8 points (52.8–59.2%); IPSS-QoL by 2.3–2.8 points (47.9–58.3%); IIEF by 3.2–5.7 points (15.6–27.8%); PSA by 1.5–2.0 ng/mL (15.6–28.5%); and prostate volume by 16.9%. Twelve (4.0%) AEs were reported; no sexual/severe AEs were observed. PAE was safe and afforded significant improvement in subjective/objective BPH parameters in patients with: prostate volumes ≥ 80 mL; prior BPH surgery/intervention; higher medical comorbidity; pre-procedural catheter-dependent urinary retention; pre-procedural renal insufficiency; and pre-procedural hematuria.

**Conclusions:**

PAE improved subjective/objective BPH parameters and sexual function. Further, it safely afforded significant improvement in subjective/objective BPH parameters in specific contexts/scenarios where current guidance for PAE is limited, including patients with larger prostates, prior BPH intervention, higher medical comorbidity, urinary retention, renal insufficiency, and hematuria. Altogether, PAE can be performed safely and effectively in several real-world contexts in which formal guidance for PAE is limited, and where it may offer unique advantages compared to surgical therapies.

**Trial registration:**

Clinicaltrials.gov, NCT03052049. Registered 6 September 2017, https://clinicaltrials.gov/study/NCT03052049; Clinicaltrials.gov, NCT02026908. Registered 10 June 2019, https://cdn.clinicaltrials.gov/large-docs/08/NCT02026908/Prot_SAP_000.pdf.

## Background

Benign prostatic hyperplasia (BPH) and associated lower urinary tract symptoms (LUTS) significantly hamper quality-of-life and carry significant personal and societal costs [21, 31, 35]. Though transurethral resection of the prostate (TURP) once represented the gold-standard for medication-refractory BPH, it is associated with high rates of erectile dysfunction (ED), retrograde ejaculation, and other adverse events (AEs) [30]. Despite the emergence of several minimally invasive surgical therapies (MISTs), many are associated with key limitations [33]. Furthermore, there exist many real-world patient factors and scenarios for which the American Urological Association (AUA) guidelines recommend few therapeutic options beyond medical therapy.

Prostate artery embolization (PAE) has emerged as a minimally invasive therapy which affords improvement in BPH symptoms and objective urinary parameters with a favorable AE profile [1, 15, 26, 29]. While PAE was recently included in the AUA Guidelines, guidance on its specific use-cases is limited [12, 33].

This study examined the safety and efficacy of PAE across real-world patient scenarios where AUA guidance for PAE is limited. Specifically, this analysis sought to understand (1) impact of PAE on sexual function, (2) safety and efficacy of PAE across prostate sizes, (3) effect of medical comorbidity on safety and efficacy of PAE, (4) safety and efficacy of PAE in the salvage setting, (5) safety and efficacy of PAE in those with catheter-dependent urinary retention, (6) safety and efficacy of PAE in those with renal insufficiency, and (7) PAE’s impact on hematuria.

## Methods

### Patient selection and study design

This single-center investigation included patients ≥ 45 years of age who underwent PAE for BPH refractory to/contraindicated for medical treatment from January 2015 until December 2024. This included patients who underwent PAE as part of two separate open-label, non-randomized clinical trials (ClinicalTrials.gov; NCT03052049 and NCT02026908), as well as patients who underwent PAE outside formal trials—appropriate institutional review board (IRB) approval was obtained. Of note, inclusion/exclusion criteria for clinical-trial and non-trial patients were identical, with few exceptions: (1) patients with history of prior prostatic surgery/procedure were excluded from clinical trial participation but could be treated off-trial, and (2) trial patients were required to undergo urodynamic testing to demonstrate baseline peak urinary flow rate (Q_max_) ≤ 12 mL/s and post-void residual ≥ 125 mL, whereas off-trial patients were not mandated to undergo urodynamic testing prior to PAE (despite many having undergone such testing as part of their BPH workup). Complete inclusion/exclusion criteria can be found on ClinicialTrials.gov.

Patients were evaluated at screening and 1/3/6/12 months post-procedure regarding (1) subjective BPH scores, including International Prostate Symptom Score (IPSS), IPSS Quality of Life (IPSS-QoL), and BPH Impact Index (BPH-II); and (2) subjective sexual function scores, including the International Index of Erectile function (IIEF) and Male Sexual Health Questionnaire for Ejaculatory Function Short Form (MSHQ-EjD-SF). IPSS results were sub-divided into voiding (i.e., IPSS-v; intermittency, weak stream, straining, and incomplete emptying) or storage symptoms (i.e., IPSS-s; frequency, urgency, and nocturia). Additionally, patients were screened for urinary retention requiring indwelling or frequent (i.e., at least daily) intermittent catheterization, renal insufficiency (effective glomerular filtration rate [eGFR] < 60 mL/min/1.73 m^2^ > 3 months, pursuant with AUA guidelines), and gross hematuria; at follow-up, persistent or resolution of urinary retention and hematuria was assessed [3]. In trial patients, objective BPH laboratory parameters—including Q_max_, PVR, and PSA—were assessed at screening and follow-up. Prostate MRIs and/or abdominopelvic CTs were used to calculate prostate volume [33]. Clinical success was defined as improved IPSS (≥ 25% reduction) or IPSS-QoL (≥ 1 point reduction) [27, 32]. AEs were graded using the Society of Interventional Radiology (SIR) classification system [3].

Finally, hospital length-of-stay and procedural radiation dose were recorded.

### Procedural details and technique

PAE was performed following standard techniques [13, 24]. This included on-table computed tomography angiography (CTA) via dedicated on-table CT in the majority of patients, and cone-beam computed tomography (CBCT) in a minority of earlier patients. In all, embolization was performed via femoral arterial access and using 300–500 µm microspheres. Bilateral pelvic side embolization was performed in all patients, except in cases where atherosclerosis or tortuosity precluded access to the prostate artery(s) of one pelvic side, in which unilateral embolization was performed.

### Statistical analyses

Subjective/objective BPH parameters, sexual function scores, and prostate volume were compared to baseline using one-sample (paired) *t* tests when the number of paired samples (*n*) was ≥ 25; when *n* was < 25, non-parametric Wilcoxon signed-rank tests were used, consistent with standard biomedical statistical practices recognizing that normality assumptions for parametric tests in small sample sizes (*n* < 20–30) increases the risk of Type 1 errors [18]. These results were additionally stratified across key variables, and sub-analyses were performed to compare results between sub-groups using two-sample (unpaired) *t* tests when n in both sub-groups was ≥ 25; when *n* was < 25 in either sub-group, Wilcoxon rank-sum tests were used. Statistical significance was defined as a *p* value < 0.05. Specifically, results were stratified and sub-analyzed by (1) pre-procedural prostate volume (< 80 mL versus ≥ 80 mL; small (< 30 mL) versus average (30–80 mL) versus large (80–150 mL) versus very large (> 150 mL)); (2) medical comorbidity at baseline (Charlson Comorbidity Index [CCI] ≥ 4, or “non-index patients,” versus CCI < 4, or “index” patients); (3) prior BPH surgery/intervention versus treatment-naïve; (4) whether patients demonstrated catheter-dependent urinary retention pre-procedure; (5) whether patients demonstrated renal insufficiency pre-procedure; (6) whether patients demonstrated gross hematuria pre-procedure [9, 33]. Laboratory parameters for BPH were not routinely obtained for off-trial patients. Also, patients who underwent prior BPH surgeries/interventions were excluded from the clinical trials. As such, analyses/sub-analyses statistically comparing Q_max_, PVR, and PSA were not performed amongst/between these specific groups/sub-groups where data was not collected.

To assess for inherent baseline differences between clinical-trial and non-trial patients, baseline IPSS, IIEF, and prostate volume were compared between these groups using two-sample (unpaired) *t* tests when *n* in both sub-groups was ≥ 25; if *n* was < 25 in either group, Wilcoxon rank-sum tests would have been used. Additionally, baseline frequencies of renal insufficiency, high medical comorbidity (i.e., CCI > 4), and urinary retention were compared between these groups using chi-squared tests; if *n* was ≤ 5 in either group, Fisher’s tests were used, consistent with standard biomedical statistical practices recognizing that utilizing parametric tests with expected frequencies < 5 increases the risk of Type 1 errors [19].

Patient attrition was assessed at 12-month follow-up as a percentage of those truly lost to follow-up, excluding those administratively censored (i.e., patients who had not reached 12 months post-PAE by the time of data analysis). To assess for inherent baseline differences between responders and eligible non-responders, baseline IPSS, IIEF, prostate volume, and PVR were compared between these groups using two-sample (unpaired) *t* tests; if *n* was < 25 in either group, Wilcoxon rank-sum tests would have been used. Additionally, baseline frequencies of renal insufficiency, high medical comorbidity (i.e., CCI > 4), prior BPH intervention, and urinary retention were compared between these groups using chi-squared tests; if *n* was ≤ 5 in either group, Fisher’s tests were used.

AEs were summarized overall and across the sub-groups described above. Given the low frequency of AEs, statistical comparison between these sub-groups was not performed.

## Results

### Baseline characteristics

Three hundred two patients were included (Table 1). Bilateral pelvic-side embolization was achieved in 297 patients (98.3%), while unilateral embolization was performed in 5 patients (1.7%) in whom atherosclerosis or tortuosity precluded bilateral embolization; in all 5 cases of unilateral embolization, the treated prostatic side was noted to supply both sides of the prostate, presumably related to chronic compensatory changes. One hundred seventy-one patients (62.8%) had prostate volumes ≥ 80 mL. One hundred eighty-three (60.6%) were medically co-morbid, “non-index” patients. Twenty-three (7.6%) had previously undergone prior BPH surgery/intervention. Thirty-eight (12.6%) required indwelling/frequent intermittent catheterization; 77 (25.5%) demonstrated renal insufficiency; and 9 (3.0%) demonstrated gross hematuria.

**Table 1:** Baseline characteristics for 302 total included patients with BPH who underwent PAE

Baseline characteristics of included patients (*n* = 302)	
**Characteristic**	**Value**
Age, mean (years)	67.8
Ethnicity, count (%)	
Caucasian	249 (82.4%)
Asian	26 (8.6%)
Hispanic	13 (4.3%)
African-American	11 (3.6%)
Other	1 (0.3%)
Length of BPH symptoms, mean (months)	81.9
Prostate volume, mean (mL)	102.6
PV < 80 mL, count (%)	171 (62.9%)
PV ≥ 80 mL, count (%)	101 (37.1%)
Cr, mean (mg/dL)	1.1
eGFR, mean (< 60 mL/min/1.73m^2^)	
Renal insufficiency, count (%)	51 (16.9%)
INR, mean	1.2
PSA, mean (ng/mL)	5.5
% free PSA, mean	24.5
Gross hematuria, count (%)	9 (3.0%)
Urinary retention requiring indwelling/intermittent catheterization, count	38 (12.6%)
Prior BPH treatment/surgery, count (%)	23 (7.6%)
TURP	10 (3.3%)
Prostatic urethral lift (PUL; UroLift)	6 (2.0%)
Water vapor thermal therapy (WVTT; Rezum)	5 (1.7%)
Photoselective vaporization of the prostate (PVP) with GreenLight	3 (1.0%)
Transurethral needle ablation (TUNA)	2 (0.7%)
Holmium laser enucleation of the prostate (HoLEP)	1 (0.3%)
PVP with PlasmaButton	1 (0.3%)
Robotic waterjet therapy (RWT; Aquablation)	1 (0.3%)
High medical comorbidity (i.e. CCI ≥ 4), count (%)	183 (60.6%)

### Changes in clinical parameters following PAE

**Fig. 1:**
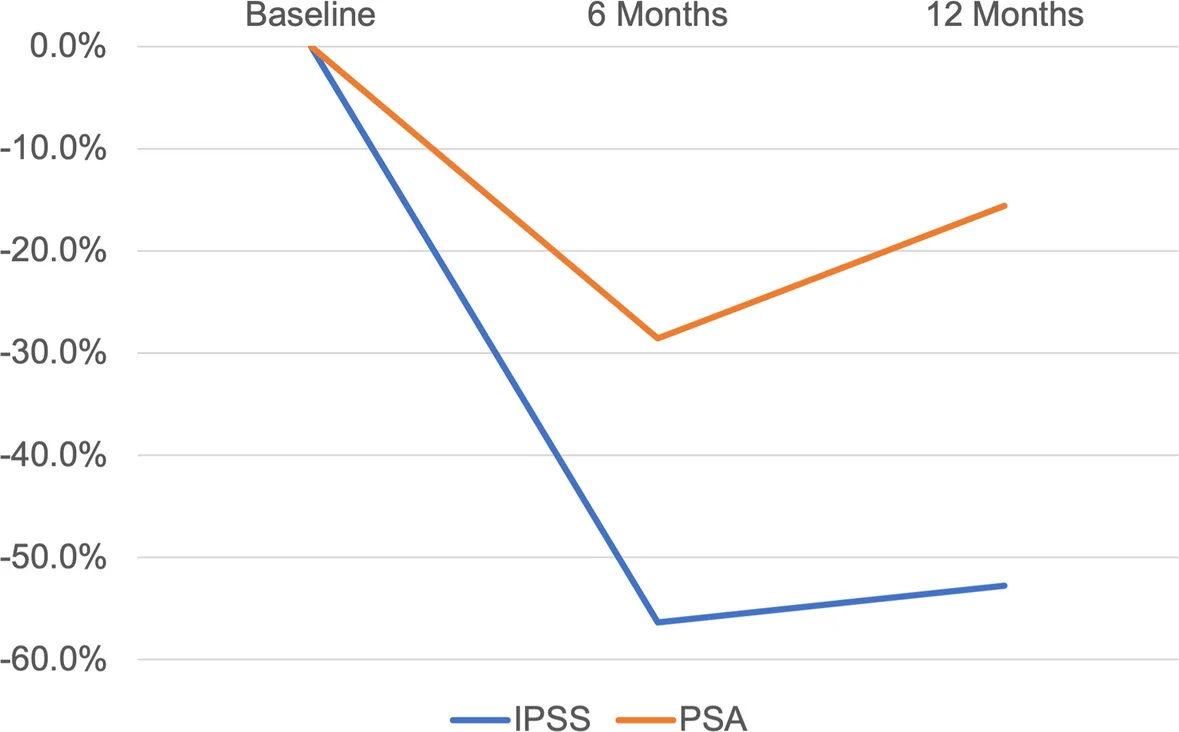
IPSS and PSA pre- and post-PAE. Changes in IPSS and PSA from baseline to 6- and 12-month follow-up

Overall, at each point, PAE was associated with significant improvement in each symptom score assessed (Table 2). This included both IPSS-s and IPSS-v at all points (Table 2). The average decrease in IPSS was 56.4% at 6 months and 52.8% at 12 months after PAE (Fig. 1; Table 2). Clinical success was achieved in 186 of 199 patients (93.4%) at 6-month follow-up and 151 of 162 patients (93.2%) at 12-month follow-up.

**Table 2:** Changes in BPH symptom scores, objective BPH parameters, and sexual function/symptom scores following PAE. For a given parameter, to compare baseline and follow-up, a paired *t* test was used, except in cases where *n* was < 25, in which case a Wilcoxon signed-rank test was used. (*) denotes a *p* value < 0.05. (ID†) denotes insufficient data for analysis

	**Changes in clinical parameters following PAE**														
	**Pre-PAE**			**1-month follow-up**			**3-month follow-up**			**6-month follow-up**			**12-month follow-up**		
	**X ± SD**	**P-Val**	***n***	**X ± SD**	**P-Val**	***n***	**X ± SD**	**P-Val**	***n***	**X ± SD**	**P-Val**	***n***	**X ± SD**	**P-Val**	***n***
**IPSS**	23.3 ± 6.1	N/A	292	11 ± 6.8	< 0.001*	223	9.5 ± 6.9	< 0.001*	210	9.9 ± 7	< 0.001*	193	10.7 ± 6.9	< 0.001*	158
**IPSS-QoL**	4.8 ± 1	N/A	299	2.5 ± 1.6	< 0.001*	224	2.1 ± 1.6	< 0.001*	214	2 ± 1.6	< 0.001*	198	2.2 ± 1.6	< 0.001*	161
**IIEF**	20.5 ± 9.2	N/A	178	23.7 ± 7.8	< 0.001*	132	24.2 ± 7.3	< 0.001*	128	25 ± 6.5	< 0.001*	111	26.2 ± 5.3	< 0.001*	89
**BPH-II**	8.2 ± 2.7	N/A	286	3.6 ± 2.9	< 0.001*	216	2.6 ± 2.9	< 0.001*	205	2.5 ± 2.8	< 0.001*	186	2.7 ± 2.9	< 0.001*	151
**MSHQ-Q1**	3.4 ± 1.5	N/A	246	3.7 ± 1.4	0.0075*	179	3.7 ± 1.4	0.36	169	3.8 ± 1.4	0.12*	155	3.8 ± 1.3	0.25	128
**MSHQ-Q2**	2 ± 1.5	N/A	245	2.7 ± 1.7	< 0.001*	180	2.8 ± 1.5	< 0.001*	169	2.7 ± 1.5	< 0.001*	155	2.7 ± 1.5	< 0.001*	128
**MSHQ-Q3**	2 ± 1.5	N/A	244	2.6 ± 1.6	< 0.001*	177	2.6 ± 1.5	< 0.001*	167	2.5 ± 1.4	0.0035*	153	2.6 ± 1.5	0.0069*	128
**MSHQ-Q4**	2.5 ± 1.7	N/A	235	1.9 ± 1.7	< 0.001*	176	1.8 ± 1.5	< 0.001*	162	1.7 ± 1.5	< 0.001*	147	1.6 ± 1.5	< 0.001*	121
**Qmax**	6.7 ± 6.2	N/A	71	13.2 ± 8.4	< 0.001*	62	15.9 ± 12.7	< 0.001*	62	8.7 ± 4.1	0.035*	7	10.5 ± 10	0.020*	42
**PVR**	156.6 ± 144.1	N/A	70	118.6 ± 135.8	0.012*	63	130 ± 114.6	0.32	60	101.1 ± 113.1	< 0.001*	60	133.9 ± 169.4	0.32	42
**Prostate Volume**	97.1 ± 53.0	N/A	272	ID†	ID†	1	ID†	ID†	1	84.1 ± 38.3	< 0.001*	57	ID†	ID†	1
**PSA**	5.1 ± 4.4	N/A	71	13.1 ± 13	0.10	4	6.3 ± 7.1	0.20	4	3.6 ± 3.6	< 0.001*	60	4.2 ± 3.5	0.0033*	48
**IPSS-s**	10.2 ± 2.9	N/A	292	5.4 ± 3.1	< 0.001*	223	4.7 ± 3.3	< 0.001*	210	4.2 ± 3	< 0.001*	193	4.9 ± 3	< 0.001*	158
**IPSS-v**	12.9 ± 4.5	N/A	292	4.4 ± 4	< 0.001*	223	4.1 ± 4.6	< 0.001*	210	3.5 ± 3.8	< 0.001*	193	3.5 ± 2.7	< 0.001*	158

PAE was associated with significant improvement in subjective sexual function at nearly all follow-up time-points (Table 2). Total IIEF scores were significantly improved from baseline at each follow-up time-point. Scores for MSHQ-EjD-SF Question 2 (“How would you rate the strength or force of your ejaculation?”), Question 3 (“How would you rate the amount or volume of semen or fluid when you ejaculate?”), and Question 4 (“If you have had any ejaculation difficulties or have been unable to ejaculate, have you been bothered by this?”) significantly improved at each follow-up time-point. Scores for MSHQ-EjD-SF Question 1 (“How often have you been able to ejaculate or ‘cum’ when having sexual activity?”) improved at 1-month follow-up. At no follow-up time-points did sexual function significantly worsen for any of the parameters assessed.

PAE was associated with significant improvement in objective laboratory parameters at nearly all follow-up time points (Table 2). Specifically, there were significant improvements in Q_max_ at all time points, in PVR at 1 and 6 months, and in PSA at all points where PSA data was collected. The average decrease in PSA was 28.5% at 6 months and 15.6% at 12 months post-PAE (Fig. 1; Table 2). Finally, PAE was associated with a significant reduction in prostate volume at 6-month follow-up, with an average decrease in prostate volume of 16.9% (*p* < 0.001; Table 2).

Among 38 patients with pre-procedural urinary retention requiring indwelling or frequent straight catheterization, 29 (76.3%) achieved complete resolution of their catheter dependence following PAE. Factoring in two additional patients who achieved decreased frequency of intermittent catheterization, 81.6% achieved improvement or resolution of their catheter dependence post-PAE.

Sub-analyses across key patient factors and scenarios outlined above can be found in Supplemental Tables 1-6.

### Adverse events and other results

At 12-month follow-up, excluding those who had not reached 12 months post-PAE by study end, the non-response rate was 33.3%, 36.9%, and 41.4% for IPSS, IIEF, and PVR data respectively. Analyses assessing baseline differences between clinical-trial versus non-trial patients and responders versus non-responders at follow-up can be found in Tables 3 and 4.

**Table 3:** Assessment for inherent baseline differences between clinical-trial and non-trial patients. Baseline IPSS, IIEF, and prostate volume were compared between these groups using two-sample (unpaired) t-tests when n in both sub-groups was ≥ 25; if *n* was < 25 in either group, Wilcoxon rank-sum tests would have been used. Additionally, baseline frequencies of renal insufficiency, high medical comorbidity (i.e., CCI > 4), and urinary retention were compared between these groups using chi-squared tests; if *n* was ≤ 5 in either group, Fisher’s tests were used. (*) denotes a *p* value < 0.05

Baseline characteristics between clinical-trial and non-trial patients			
	**X ± SD**	***P***-Val	**n**
**IPSS**	23.3 ± 6.1	N/A	292
Trial	24.3 ± 6.3	0.15	68
Non-trial	23.0 ± 6.0		224
**IIEF**	20.5 ± 9.2	N/A	178
Trial	21.6 ± 8.9	0.36	48
Non-trial	20.2 ± 9.4		130
**Prostate volume**	97.1 ± 53.0	N/A	272
Trial	95.6 ± 51.6	0.41	71
Non-trial	105.6 ± 55.9		201
	**Frequency**	***P***-Val	**n**
**Renal insufficiency**	16.9%	N/A	51
Trial	11.3%	0.089	7
Non-trial	23.5%		44
**Medically comorbid (“non-index”)**	60.6%	N/A	183
Trial	77.5%	0.0037*	55
Non-trial	55.4%		128
**Urinary retention**	12.6%	N/A	38
Trial	11.3%	0.71	8
Non-trial	13.0%		30

**Table 4:** Assessment for inherent baseline differences between responders and eligible non-responders (i.e., excluding those who had not reached 12-months post-PAE by the time of data analysis), where response was defined as participating in IPSS/IIEF surveys and PVR testing at 12-month follow-up. Baseline IPSS, IIEF, prostate volume, and PVR were compared between these groups using two-sample (unpaired) *t* tests; if *n* was < 25 in either group, Wilcoxon rank-sum tests would have been used. Additionally, baseline frequencies of renal insufficiency, high medical comorbidity (i.e., CCI > 4), prior BPH intervention, and urinary retention were compared between these groups using chi-squared tests; if *n* was ≤ 5 in either group, Fisher’s tests were used. (*) denotes a *p* value < 0.05

Baseline characteristics between responders and non-responders (12-month follow-up)									
	**Response/non-response to IPSS survey at 12-months**			**Response/non-response to IIEF survey at 12-months**			**Response/non-response to PVR testing at 12-months**		
	**X ± SD**	**P-Val**	**n**	**X ± SD**	**P-Val**	**n**	**X ± SD**	**P-Val**	**n**
**IPSS**	23.3 ± 6.1	N/A	292	23.3 ± 6.1	N/A	292	23.3 ± 6.1	N/A	292
Responders	23.3 ± 5.9	0.97	158	24.1 ± 6.1	0.18	105	23.1 ± 6.2	0.11	38
Non-Responders	23.3 ± 6.2		79	21.8 ± 6.2		52	25.6 ± 6.0		29
**IIEF**	20.5 ± 9.2	N/A	178	20.5 ± 9.2	N/A	178	20.5 ± 9.2	N/A	178
Responders	21.8 ± 8.3	0.12	105	21.8 ± 8.3	0.42	105	20.9 ± 9.2	0.52	26
Non-responders	18.7 ± 11.0		37	18.6 ± 9.8		52	22.6 ± 8.9		21
**Prostate volume**	97.1 ± 53.0	N/A	272	97.1 ± 53.0	N/A	272	97.1 ± 53.0	N/A	272
Responders	99.5 ± 51.8	0.30	72	97.8 ± 50.6	0.50	53	108.3 ± 51.0	0.24	39
Non-responders	87.2 ± 38.2		15	112.5 ± 52.4		7	80.1 ± 48.2		28
**PVR**	156.6 ± 144.1	N/A	70	156.6 ± 144.1	N/A	70	156.6 ± 144.1	N/A	70
Responders	146.3 ± 141.7	0.45	61	156.6 ± 150.2	0.34	42	160.6 ± 124.0	0.79	41
Non-responders	208.2 ± 183.4		6	208.0 ± 85.2		4	150.9 ± 170.8		29
	**Frequency**	**P-Val**	**n**	**Frequency**	**P-Val**	**n**	**Frequency**	**P-Val**	**n**
**Renal insufficiency**	16.9%	N/A	51	16.9%	N/A	51	16.9%	N/A	51
Responders	16.0%	0.38	25	10.6%	0.88	11	15.0%	0.23	6
Non-responders	20.3%		16	11.5%		6	3.6%		1
**Medically comorbid (“non-index”)**	60.6%	N/A	183	60.6%	N/A	183	60.6%	N/A	183
Responders	71.6%	0.0011*	113	80.0%	0.025*	84	73.2%	0.35	30
Non-responders	49.6%		39	61.5%		32	82.4%		24
**Prior BPH intervention**	7.6%	N/A	23	7.6%	N/A	23	7.6%	N/A	23
Responders	7.6%	1.00	12	6.7%	0.75	7	0.0%	1.00	0
Non-responders	7.6%		6	7.7%		4	0.0%		0
**Urinary retention**	12.6%	N/A	38	12.6%	N/A	38	12.6%	N/A	38
Trial	10.1%	0.51	16	11.4%	0.27	12	14.6%	0.46	6
Non-trial	12.7%		10	7.7%		4	6.9%		2

Twelve patients (4.0%) experienced AEs, all of which were mild (10; 3.3%) or moderate (2; 0.7%); no severe or life-threatening/disabling AEs were observed. Specifically, these AEs included hematuria (4; 1.3%); UTI requiring antibiotics (3; 1.0%); urinary retention requiring ≤ 3 straight catheterizations (3; 1.0%); urinary retention requiring indwelling catheterization (1; 0.3%) for approximately one month; and hematospermia (1; 0.3%) [17].

Mean hospital length-of-stay was 0 days; all patients were treated as outpatients. The average procedural radiation dose was 3470 milligray.

## Discussion

In over 300 patients, PAE was associated with significant improvement in subjective/objective BPH parameters at all points with very few exceptions, and significantly improved sexual function. Specifically, across follow-up, the following average improvements in subjective and objective BPH parameters were observed in the present analysis, consistent with those reported in prior analyses of PAE: IPSS by 12.2–13.8 points (50.6–57.6%); IPSS-QoL by 2.5–2.8 points (52.1–58.3%); BPH-II by 4.7–5.9 points (54.8–67.7%); Q_max_ by 3.5–8.0 mL/s (64.2–150.4%); PVR by 9.5–29.1 mL (6.1–18.6%); PSA by 1.5–2.0 ng/mL (15.6–28.5%); and prostate volume by 21.5 mL (16.9%) [20, 26]. This was accomplished with an extremely favorable AE profile, with no sexual or severe AEs.

### PAE and sexual function

At no points did sexual function significantly worsen for any of the parameters assessed, nor were any sexual AEs observed. These results are consistent with many analyses demonstrating preservation of sexual function with PAE [2, 5, 20]. This contrasts TURP, which is limited by its relatively high rates of retrograde ejaculation, and certain MISTs which may risk worsening sexual function [4, 20]. Interestingly, PAE was actually associated with significant *improvement* in sexual function at all points, except for MSHQ-EjD-SF Question 1 at 3-, 6-, and 12-month follow-up, which substantiates the findings of several other analyses demonstrating improved sexual function after PAE [8, 22, 28, 34]. The 2026 AUA guidelines now recognizes PAE's favorable sexual function profile, and the present data further substantiate this assessment. Given these results, PAE should be recommended for patients seeking to preserve, and possibly improve, their sexual function.

### Pre-procedural prostate volume effects

PAE was safe and afforded significant improvement in subjective BPH parameters, objective BPH parameters, and sexual function, in patients with prostate volumes ≥ 80 mL compared to patients with prostate volumes < 80 mL. These levels of improvement were comparable between the two groups except at a few points where the sub-group of patients with prostate volumes of ≥ 80 mL saw slightly more favorable results for certain BPH parameters. These findings are congruent with the published literature, which has shown that PAE is very safe and effective in patients with larger prostates, but likely affords diminishing returns in smaller prostates [25]. Additionally, the results from the present analysis among patients with “very large” prostates (i.e., ≥ 150 mL) demonstrating significant improvement in every subjective and objective BPH parameter at nearly every point with preservation (and, at several points, improvement) in sexual function reinforces the fact that PAE is not limited by an upper bound on prostate size [25]. The 2026 AUA guideline now recommends PAE for prostates ≥50 mL without an upper volume limit, in contrast to several MISTs that are limited to prostates 30–80 mL [33]. Furthermore, unlike many other MISTs, PAE can be safely and effectively performed in patients with a large median lobe [25]. The present data reinforce this recommendation and extend it to very large prostates (>150 mL), where PAE afforded significant improvement across all parameters without increased adverse events.

### Medical comorbidities

PAE was not only safe, but similarly efficacious in improving subjective/objective BPH parameters, prostate volume, and sexual function in medically-comorbid, “non-index” patients compared to less medically comorbid, “index” patients, except at 3 months for IPSS and at 1/3 months for IPSS-QoL, where “non-index” patients saw significantly *greater* improvement than “index” patients. Specifically, significant improvements were seen in these variables at nearly all points amongst “non-index” patients. These findings substantiate several prior studies illustrating PAE’s safety and efficacy in multi-morbid patients [7, 36]. The 2026 AUA guideline now includes PAE as a treatment option for patients at high risk of bleeding or those requiring anticoagulation/antiplatelet therapy during BPH procedures [33]. However, the guideline's definition of "medically complicated" remains narrow, focusing primarily on bleeding risk, and does not fully capture the spectrum of real-world medical comorbidities that may preclude surgical intervention. In practice, non-index patients are often excluded from surgery. PAE has the advantage of being performed under moderate/local sedation, obviating the need for more intensive and risky general and/or regional anesthesia. The results of the present study extend the guideline recommendation and suggest that PAE should be offered to patients with broader comorbidity profiles beyond bleeding risk alone.

### Salvage PAE

Improvements in both BPH symptoms and sexual function were not significantly different between treatment-naïve patients and “salvage” patients who had undergone prior BPH surgery/intervention at any points. This was accomplished safely in these “salvage” patients, with a comparable, favorable AE profile. Though limited data exists surrounding PAE in the salvage setting, some data have also shown that PAE represents a safe and effective treatment in patients who have undergone prior MIST, without negatively impacting erectile/ejaculatory function [14].

These results support using PAE in patients who have previously undergone intervention for BPH [10, 11, 16]. Given that PAE employs a different mechanism of action than other BPH interventions (i.e., endovascular, versus transurethral), PAE may, in certain patients and post-surgical anatomies, be uniquely poised to minimize risk of AEs and maximize therapeutic efficacy.

### Pre-procedural urinary retention

Over 75% with catheter-dependent urinary retention pre-PAE saw complete resolution in their catheter dependence following PAE, with several additional patients seeing significant improvement in the frequency of intermittent catheterization post-PAE. PAE was very safe and no less effective in the treatment of subjective/objective BPH parameters and sexual function in patients with pre-procedural catheter dependence for urinary retention; the only exception was at 1-month follow-up for IPSS-QoL, where those with pre-procedural catheter-dependent urinary retention saw significantly greater improvement, and at 3-month follow-up for Q_max_, where those without pre-procedural catheter-dependent urinary retention saw significantly greater improvement.

The 2026 AUA guideline recommends resective technologies for catheter-dependent patients, taking into consideration patient comorbidities. However, PAE offers a minimally invasive alternative for patients with catheter-dependent retention who are not surgical candidates, who have high surgical risk, or who require continued anticoagulation — consistent with AUA 2026 guidance for medically complex patients [33]. In the present analysis, the majority of patients with catheter-dependent retention achieved complete resolution of catheter dependence following PAE, with a favorable adverse event profile. These results support PAE as an effective option in this population, particularly when resective surgery is not feasible or desired [6, 7, 23].

### Pre-procedural renal insufficiency

PAE afforded safety, significant improvement in subjective BPH parameters at all points which was statistically non-inferior to the degree of improvements compared to those without renal insufficiency at the majority of points, except in IPSS-QoL at 3 months where the group without renal insufficiency saw slightly greater improvement. PAE afforded statistically non-inferior degrees of improvement in sexual function compared to those without renal insufficiency at all follow-ups. Furthermore, PAE afforded significant improvement in objective BPH parameters at most points, which was statistically non-inferior to the degree of improvements compared to those without renal insufficiency at all points, including at 1, 3, and 6 months for PVR and in PSA at 6 months where those with renal insufficiency actually saw significantly greater relative improvement. The AUA guidelines currently recommend surgical therapy (rather than medical therapy alone) for patients with renal insufficiency secondary to BPH [33]. While further research is needed, the present results demonstrate that PAE is safe and effective in this population and support its consideration as an option alongside surgical therapies for patients with BPH-related renal insufficiency.

### Pre-procedural hematuria

AUA guidelines recognizes PAE as an option for patients with hematuria of prostatic origin, regardless of prostatic size [33]. In this analysis, all 9 patients with pre-procedural hematuria saw resolution of their hematuria following PAE, further substantiating this guideline recommendation. Additionally, for patients at high risk of bleeding, the 2026 AUA guideline now includes PAE alongside HoLEP, ThuLEP, and PVP as a treatment option [33]. The present outcomes, achieved with a 0% transfusion rate consistent with other analyses [25], reinforce PAE's suitability in this population.

### Limitations

While inclusion of both clinical-trial and non-trial patients introduces potential selection bias, apart from a higher frequency of medically comorbid “non-index” patients in the clinical-trial group compared to the non-trial group, there was otherwise no significant difference between these groups with regards to baseline IPSS, IIEF, or prostate volume, nor to baseline frequencies of renal insufficiency or urinary retention. These findings suggest that clinical-trial and non-trial patients were ultimately relatively comparable.

There was moderate attrition at follow-up. Perhaps unsurprisingly, there were significantly higher frequencies of higher-comorbidity patients among those who completed 12-month IPSS/IIEF follow-up—possibly related to more frequent overall healthcare system engagement. Notably, clinical-trial patients were explicitly instructed to follow-up at 1, 3, 6, and 12 months, unlike off-trial patients who were merely encouraged to do so. While these factors could introduce bias, there were no differences in baseline IPSS, IIEF, PVR, nor in baseline frequencies of renal insufficiency, prior BPH intervention, or urinary retention between those who responded to IPSS/IIEF questionnaires or who presented for PVR assessment at 12-month follow-up, suggesting relatively high baseline equipoise between responders and non-responders.

The present study was also both strengthened and limited by the large number of sub-analyses performed, which increases the risk of Type 1 errors.

Finally, this study did not follow patients beyond 12 months post-PAE; longer-term data would be welcome to further assess the durability of PAE’s benefits in these key patient populations and clinical scenarios.

## Conclusions

Across various subgroups, PAE was associated with significant improvement in subjective/objective BPH parameters at all follow-ups with few exceptions. The present study offers insight into real-world scenarios and patient factors that reinforce and extend the 2026 AUA guideline recommendation for PAE — including several contexts where patients have historically had limited therapeutic options. Specifically, the present study showed PAE to be safe and effective in patients with hopes to preserve or even improve sexual function, history of prior BPH intervention, larger gland sizes, higher comorbidity, catheter-dependent urinary retention, renal insufficiency, and hematuria.

## Data Availability

The datasets used and/or analyzed during the current study are available from the corresponding author on reasonable request.

## Abbreviations

5-ARIs: 5-Alpha-reductase inhibitors
AE(s): Adverse event(s)
AUA: American Urological Association
BPH: Benign prostatic hyperplasia
BPH-II: BPH Impact Index
CBCT: Cone-beam computed tomography
CCI: Charlson Comorbidity Index
CT: Computed tomography
CTA: Computed tomography angiography
ED: Erectile dysfunction
eGFR: Estimated glomerular filtration rate
IIEF: International Index of Erectile Function
IPSS: International Prostate Symptom Score
IPSS-QoL: IPSS Quality of Life
IPSS-s: IPSS storage symptoms
IPSS-v: IPSS voiding symptoms
IRB: Institutional review board
LUTS: Lower urinary tract symptoms
MISTs: Minimally-invasive surgical therapies
MRI: Magnetic resonance imaging
MSHQ-EjD-SF: Male Sexual Health Questionnaire for Ejaculatory Dysfunction Short Form
PAE: Prostate artery embolization
PSA: Prostate-specific antigen
PVR: Post-void residual
Q_max_: Peak urinary flow rate
QoL: Quality of life
SIR: Society of Interventional Radiology
TURP: Transurethral resection of the prostate
UTI: Urinary tract infection

## References

[1] Abt D, Hechelhammer L, Müllhaupt G, et al. Comparison of prostatic artery embolisation (PAE) versus transurethral resection of the prostate (TURP) for benign prostatic hyperplasia: randomised, open label, non-inferiority trial. BMJ. 2018;361:k2338. 10.1136/bmj.k2338 PMC6006990 29921613

[2] Al Rawashdah SF, Pastore AL, Velotti G, et al. Sexual and functional outcomes of prostate artery embolisation: a prospective long-term follow-up, large cohort study. Int J Clin Pract. 2020;74(3):e13454. 10.1111/ijcp.13454 31769906

[3] Baerlocher MO, Nikolic B, Sze DY. Adverse event classification: clarification and validation of the Society of Interventional Radiology specialty–specific system. J Vasc Interv Radiol. 2023;34(1):1–3. 10.1016/j.jvir.2022.10.011 36244632

[4] Bebi C, Turetti M, Lievore E, et al. Sexual and ejaculatory function after holmium laser enucleation of the prostate and bipolar transurethral enucleation of the prostate: a single-center experience. Int J Impot Res. 2022;34(1):71–80. 10.1038/s41443-020-00366-8 33082545

[5] Bhatia S, Acharya V, Jalaeian H, et al. Effect of prostate artery embolization on erectile function—a single center experience of 167 patients. J Sex Med. 2022;19(4):594–602. 10.1016/j.jsxm.2022.01.006 35184995

[6] Bhatia S, Bhatia A, Richardson AJ, et al. Prostatic artery embolization: mid- to long-term outcomes in 1,075 patients. J Vasc Interv Radiol. 2025;36(3):456–66. 10.1016/j.jvir.2024.11.002 39532156

[7] Bhatia S, Sinha VK, Kava BR, et al. Efficacy of prostatic artery embolization for catheter-dependent patients with large prostate sizes and high comorbidity scores. J Vasc Interv Radiol. 2018;29(1):78-84.e71. 10.1016/j.jvir.2017.08.022 29150394

[8] Bilhim T, Costa NV, Torres D, Pinheiro LC, Spaepen E. Long-term outcome of prostatic artery embolization for patients with benign prostatic hyperplasia: single-centre retrospective study in 1072 patients over a 10-year period. Cardiovasc Intervent Radiol. 2022;45(9):1324–36. 10.1007/s00270-022-03199-8 35778579

[9] Charlson ME, Carrozzino D, Guidi J, Patierno C. Charlson comorbidity index: a critical review of clinimetric properties. Psychother Psychosom. 2022;91(1):8–35. 10.1159/000521288 34991091

[10] Dean NS, Assmus MA, Lee MS, Guo JN, Krambeck AE. Benign prostatic hyperplasia surgical re-treatment after prostatic urethral lift a narrative review. Can Urol Assoc J. 2023;17(10):353–9. 10.5489/cuaj.8334 PMC10581727 37494319

[11] Durant AM, Moore J, Voleti S, et al. Salvage versus primary holmium laser enucleation of the prostate: trends, outcomes and safety analysis. World J Urol. 2022;40(9):2305–12. 10.1007/s00345-022-04098-w 35867143

[12] Foster HE, Dahm P, Kohler TS, et al. Surgical management of lower urinary tract symptoms attributed to benign prostatic hyperplasia: AUA guideline amendment 2019. J Urol. 2019;202(3):592–8. 10.1097/JU.0000000000000319 31059668

[13] Goyal P, Salem R, Mouli SK. Controversies in prostate artery embolization: future best practice. Semin Intervent Radiol. 2022;39(6):562–70. 10.1055/s-0042-1759701 PMC9767779 36561793

[14] Goyal P, Varadhan AK, Jenkins K, Hohlastos E, Salem R, Mouli SK. Prostatic artery embolization in patients with refractory lower urinary tract symptoms after a prior minimally invasive surgical treatment. J Vasc Interv Radiol. 2024;35(5):744–50. 10.1016/j.jvir.2024.01.026 38311293

[15] Insausti I, Sáez de Ocáriz A, Galbete A, et al. Randomized comparison of prostatic artery embolization versus transurethral resection of the prostate for treatment of benign prostatic hyperplasia. J Vasc Interv Radiol. 2020;31(6):882–90. 10.1016/j.jvir.2019.12.810 32249193

[16] Kallidonis P, Spinos T, Peteinaris A, Somani B, Liatsikos E. Salvage holmium laser enucleation of the prostate after previous interventions: a systematic review. BJU Int. 2024;133(2):141–51. 10.1111/bju.16131 37461135

[17] Katayama H, Kurokawa Y, Nakamura K, et al. Extended Clavien-Dindo classification of surgical complications: Japan Clinical Oncology Group postoperative complications criteria. Surg Today. 2016;46(6):668–85. 10.1007/s00595-015-1236-x PMC4848327 26289837

[18] Kim HY. Statistical notes for clinical researchers: Nonparametric statistical methods: 1. Nonparametric methods for comparing two groups. Restor Dent Endod. 2014;39(3):235–9. 10.5395/rde.2014.39.3.235 PMC4125590 25110650

[19] Kim HY. Statistical notes for clinical researchers: Chi-squared test and Fisher’s exact test. Restor Dent Endod. 2017;42(2):152–5. 10.5395/rde.2017.42.2.152 PMC5426219 28503482

[20] Knight GM, Talwar A, Salem R, Mouli S. Systematic review and meta-analysis comparing prostatic artery embolization to gold-standard transurethral resection of the prostate for benign prostatic hyperplasia. Cardiovasc Intervent Radiol. 2021;44(1):183–93. 10.1007/s00270-020-02657-5 33078236

[21] Lepor H. Pathophysiology of lower urinary tract symptoms in the aging male population. Rev Urol. 2005;7(Suppl 7(Suppl 7)):S3-s11. PMC1477625 16986059

[22] Maclean D, Maher B, Modi S, et al. Prostate artery embolization: a new, minimally invasive treatment for lower urinary tract symptoms secondary to prostate enlargement. Ther Adv Urol. 2017;9(8):209–16. 10.1177/1756287217717889 PMC5896862 29662545

[23] Marchi A, Pellerin O, Querub C, et al. Long-term outcomes of prostate artery embolization for acute urinary retention: an analysis of 88 patients. Diagn Interv Imaging. 2023;104(6):292–6. 10.1016/j.diii.2023.01.012 36801097

[24] Mouli S, Hohlastos E, Salem R. Prostate artery embolization. Semin Intervent Radiol. 2019;36(2):142–8. 10.1055/s-0039-1688431 PMC6531021 31123388

[25] Mouli S, Salem R, McClure TD. Prostate artery embolization for benign prostatic hyperplasia. J Urol. 2024;212(1):216–9. 10.1097/JU.0000000000003976 PMC12721628 38703386

[26] Pisco JM, Bilhim T, Costa NV, et al. Randomised clinical trial of prostatic artery embolisation versus a sham procedure for benign prostatic hyperplasia. Eur Urol. 2020;77(3):354–62. 10.1016/j.eururo.2019.11.010 31831295

[27] Pisco JM, Bilhim T, Pinheiro LC, et al. Medium- and long-term outcome of prostate artery embolization for patients with benign prostatic hyperplasia: results in 630 patients. J Vasc Interv Radiol. 2016;27(8):1115–22. 10.1016/j.jvir.2016.04.001 27321890

[28] Pisco JM, Rio Tinto H, Campos Pinheiro L, et al. Embolisation of prostatic arteries as treatment of moderate to severe lower urinary symptoms (LUTS) secondary to benign hyperplasia: results of short- and mid-term follow-up. Eur Radiol. 2013;23(9):2561–72. 10.1007/s00330-012-2714-9 23370938

[29] Ray AF, Powell J, Speakman MJ, et al. Efficacy and safety of prostate artery embolization for benign prostatic hyperplasia: an observational study and propensity-matched comparison with transurethral resection of the prostate (the UK-ROPE study). BJU Int. 2018;122(2):270–82. 10.1111/bju.14249 29645352

[30] Sagen E, Namnuan RO, Hedelin H, Nelzén O, Peeker R. The morbidity associated with a TURP procedure in routine clinical practice, as graded by the modified Clavien-Dindo system. Scand J Urol. 2019;53(4):240–5. 10.1080/21681805.2019.1623312 31156002

[31] Saigal CS, Joyce G. Economic costs of benign prostatic hyperplasia in the private sector. J Urol. 2005;173(4):1309–13. 10.1097/01.ju.0000152318.79184.6f 15758787

[32] Salem R, Hairston J, Hohlastos E, et al. Prostate artery embolization for lower urinary tract symptoms secondary to benign prostatic hyperplasia: results from a prospective FDA-approved investigational device exemption study. Urology. 2018;120:205–10. 10.1016/j.urology.2018.07.012 30036614

[33] Goueli R, Badlani GH, Welliver C, et al. Management of lower urinary tract symptoms attributed to benign prostatic hyperplasia (BPH): AUA guideline 2026. Part III: procedural/surgical management. J Urol. 2026. 10.1097/JU.0000000000005099. 42095468

[34] Sapoval M, Thiounn N, Descazeaud A, et al. Prostatic artery embolisation versus medical treatment in patients with benign prostatic hyperplasia (PARTEM): A randomised, multicentre, open-label, phase 3, superiority trial. Lancet Reg Health Eur. 2023;31:100672. 10.1016/j.lanepe.2023.100672 PMC10320610 37415648

[35] Speakman MJ, Cheng X. Management of the complications of BPH/BOO. Indian J Urol. 2014;30(2):208–13. 10.4103/0970-1591.127856 PMC3989825 24744522

[36] Zorzi F, Rossin G, Digregorio M, et al. Prostatic artery embolization in elderly comorbid patients with benign prostatic hyperplasia: Safety, efficacy, and predictive factors of clinical failure. J Pers Med. 2025. 10.3390/jpm15010023. PMC11767102 39852215

